# Identification of *miR-200a* as a novel suppressor of connexin 43 in breast cancer cells

**DOI:** 10.1042/BSR20150153

**Published:** 2015-09-29

**Authors:** Jia Ming, Yan Zhou, Junze Du, Shenghao Fan, Beibei Pan, Yinhuan Wang, Lingjun Fan, Jun Jiang

**Affiliations:** * Department of Breast, Thyroid and Pancreas Surgery, the Second Affiliated Hospital of Chongqing Medical University, Chongqing 400010, China; † Breast Disease Center, the First Affiliated Hospital of The Third Military Medical University, Chongqing 400038, China

**Keywords:** breast cancer, connexin 43 (Cx43), metastasis, micro ribonucleic acid (*miR*)*-200a*, micro ribonucleic acid (*miR*)*-1*, 3′-untranslated region (3′-UTR)

## Abstract

Both miRNAs (miRs) and connexin 43 (Cx43) were important regulators of the metastasis of breast cancer, whereas the miRs regulating Cx43 expression in breast cancer cells were still obscure. In the present study, we scanned and found *miR-1*, *miR-206*, *miR-200a*, *miR-381*, *miR-23a/b* and *miR-186* were functional suppressors of human Cx43 mRNA and protein expression. Specially, we demonstrated that only *miR-200a* could directly target the 3′-untranslated region (3′-UTR) of human Cx43 gene. Functionally, overexpression of Cx43 in MCF cells potentiated the migration activity, whereas additional *miR-200a* treatment notably prevented this effect. Finally, we demonstrated that decreased levels of *miR-200a* and elevated expression of Cx43 in the metastatic breast cancer tissues compared with the primary ones. Thus, we are the first to identify *miR-200a* as a novel and direct suppressor of human Cx43, indicating that *miR200a*/Cx43 axis might be a useful diagnostic and therapeutic target of metastatic breast cancer.

## Abstract (precis)

We demonstrated that miRNA (*miR*)*-200a* inhibited connexin 43 (Cx43) expression by directly targeting at the 3’-UTR of Cx43 gene in human breast cancer cells. The *miR-200a*/Cx43 axis might be a useful diagnostic and therapeutic target of metastatic breast cancer.

## INTRODUCTION

Breast cancer is the most common malignant tumour in women and the second leading cause of cancer-related death among women worldwide [1–2]. Many breast tumours are not completely eradicated due to relapse, which results in metastasis at later stages leading to death [3]. Breast cancer metastasis is associated with an increase in connexin 43 (Cx43) expression and intercellular exchange disorders [4–6].

Connexin proteins form the gap junction, a membrane structure between adjacent cells, mediating mutual, direct electric and chemical signal communications. There are 21 members in the connexin family, which are named according to their relative molecular mass (*M*_r_); for example, Cx43 refers to connexin with a *M*_r_ of 43000. Human breast tissues mainly express Cx26 and Cx43 [7]. Cx43 can mediate the adhesion of cancer cells with vascular endothelial cells [8], alter the cytoskeleton [9,10] and increase cell migration [11] through regulation of intercellular communications.

miRNAs (miRs) form one of the largest groups of post-transcriptional regulatory factors [12]. They had 2–8 bases at the 5′-end that could bind with the 3′-UTR of the target gene so as to repress gene translation and reduce protein expression [13]. miRs are involved in a series of biological processes including cell cycle, growth, apoptosis, differentiation and stress response [14,15]. Especially, increasing evidences indicated that miRs were important regulators of breast cancer metastasis [16–20].

However, whether the miRs would regulate the metastasis of breast cancer cells via Cx43 was still not known. In the present study, we screened the 3′-UTR of human Cx43 gene and identified *miR-200a* as a direct suppressor of Cx43. We suggested that the *miR-200a*/Cx43 axis might play an important role in the metastasis of breast cancer.

## MATERIALS AND METHODS

### Cell lines and culture conditions

The MDA-MB-231 is an aggressive breast cancer cell line that lacks the expression of estrogen receptor α (ERα), progesterone receptor (PR) and human epidermal growth factor receptor-2 (HER-2) [21]. MCF-7 is a non-aggressive breast cancer cell line that has ERα and HER-2 expression [21]. Both the MDA-MB-231 and the MCF-7 cells were purchased from the Shanghai Cell Bank of the Chinese Academy of Sciences. The cells were cultured in high-glucose Dulbecco's Modified Eagle's Medium (DMEM, Hyclone) containing 10% FBS (Gibco).

### Tissue collection

From October 1, 2012 to July 1, 2013, we collected 20 cases of primary tissues and 20 cases of pulmonary metastases of breast cancer that were pathologically diagnosed at the First Affiliated Hospital of the Third Military Medical University and the Second Affiliated Hospital of Chongqing Medical University. The age range of the patients was 32–67 years (median, 53 years) and the tumour diameter ranged from 1.5 to 4.7 cm (average, 2.8 cm). The patients did not receive pre-operative neoadjuvant chemotherapy or endocrine therapy. The primary cancer tissues were obtained in a sterile manner by modified radical mastectomy. Two to three pieces of the specimens from pulmonary metastases were obtained from metastatic patients during computed tomography-guided biopsy.

The present study was approved by the Ethics Committee of the First Affiliated Hospital of The Third Military Medical University and the Second Affiliated Hospital of Chongqing Medical University. All patient-derived tissues were obtained with their written informed consent.

### Real-time PCR

Total RNAs were extracted from tissues or cells with Trizol reagent Invitrogen) according to the manufacture's protocol. RNAs were transcribed into cDNAs using Omniscript (Qiagen). Quantitative real-time PCR was performed using the 7900HT Fast Real-Time PCR system (Applied Biosystems). The mRNA expression levels were normalized to the expression of glyceraldehyde-3-phosphate dehydrogenase (GAPDH). Reactions were done in duplicate using Applied Biosystems Taqman Gene Expression Assays and Universal PCR Master Mix. The relative expression was calculated by the 2(^−DDCt^) method. All the primers used for PCR are available upon request.

### Western blot analysis

Total proteins were extracted with cell lysis buffer. Then, 50 μg of protein was separated using SDS/PAGE (12% gel), electrotransferred to PVDF membranes (Millipore) and incubated with rabbit anti-human Cx43 antibody (1:1000, Boster) and then anti-rabbit secondary antibody (1:2500, Beyotime). Protein bands were quantified from the membrane by densitometry using the Adobe Photoshop V7.01 imaging system.

### Transwell migration assay

The MDA-MB-231 and MCF-7 cells were transfected with the Cx43 overexpression plasmids and/or miRs for 24 h and then the cell suspension (200 μl of cell suspension, 1×10^5^/ml) was sucked into the upper chamber of Transwell (PC membrane with 8.0-μm pore size). After culture for 12 h, the upper chamber was air-dried, fixed with paraformaldehyde for 15 min and stained with 0.1% crystal violet and five fields of view were randomly selected to count cells under a microscope (200×). The cell migration activity was described as the relative cell numbers of the transmitted cells.

### Molecular cloning experiments

Those experiments for plasmid construction, cell transfection, reporter gene assays and site-directed reporter gene mutation were performed as described in a previous study [22]. The wild-type (wt) or mutated human Cx43 3′-UTR sequences were subcloned into a pMIR-REPORT vector (Invitrogen) to form different reporter genes.

For the cloning of human Cx43 overexpression constructs, the total cellular RNA was extracted from MDA-MB-231 cells. According to the instructions of the PrimeScript 1st strand cDNA Synthesis Kit (TaKaRa), reverse transcription was performed to prepare the cDNA for the coding region or 3′-UTR of human Cx43.

The human Cx43 coding sequence (CDS) exons were subcloned into the pCDNA3.1 vector to construct a human Cx43 overexpression plasmid pCDNA-Cx43Δ, which could not be inhibited by *miR-200a* due to lack of 3′-UTR. The primer sequence used for human Cx43 CDS exons cloning was: F: 5′-GGACTAGTATGGGTGACTGGAGCGCCTT-3′; R: 5′-CGACGCGTCTAGATCTCCAGGTCATCAG-3′. The SpeI and MulI restriction enzyme cutting sites are underlined.

The human Cx43 CDS exons plus 3′-UTR were inserted into the pCDNA3.1 vector to construct a human Cx43 overexpression plasmid pCDNA-Cx43, which could be inhibited by *miR-200a* via the 3′-UTR. The primer sequence used for human Cx43 CDS exons plus UTR cloning was: F: 5′-GGACTAGTATGGGTGACTGGAGCGCCTT-3′; R: 5′-CGACGCGTTATGTTTATACTAAATTAAA-3′. The SpeI and MulI restriction enzyme cutting sites are underlined.

### Statistics

Data are generated as mean ± S.D. Comparisons were performed using ANOVA for multiple groups or Student's *t*test for two groups. *P*<0.05 is considered statistically significant. Values not sharing a common superscript letter differ significantly (*P*<0.05).

## RESULTS

### Prediction of potential miRs targeting in the 3′-UTR of Cx43 gene

To explore the miRs involvement in Cx43 gene expression, we screened the potential binding sites for different miRs in the 3′-UTR of human (Figure 1A), mouse (Figure 1B) and rat (Figure 1C) Cx43 genes by using an online software (http://www.microrna.org/microrna). We selected out the common miRs from those three species (Figure 1D), since those conserved miRs might be evolutionarily selected as functional suppressors. Among the conserved ones, *miR-1* and *miR-206* had been identified as direct suppressors of Cx43 in a previous study [23]. However, the regulatory roles of other conserved miRs like *miR-101a/b*, *miR-200a*, *miR-141*, *miR-381*, *miR-130a/b*, *miR-301a/b*, *miR-23a/b*, *miR-30a*, *miR-30b-5p*, *miR-30c*, *miR-30d*, *miR-30e*, *miR-186*, *miR-19a/b* and *miR-144* in Cx43 gene expression were still not identified.

**Figure 1:**
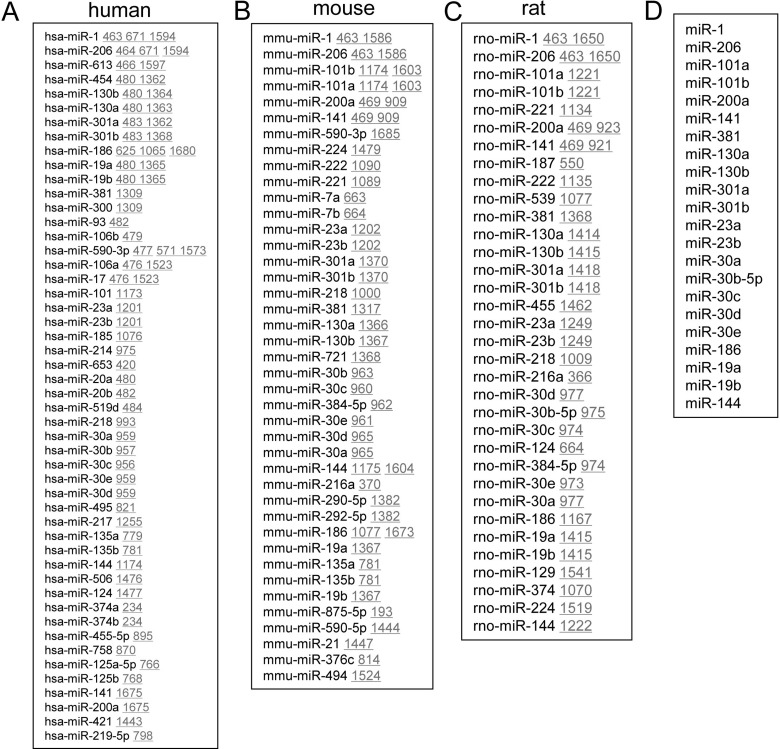
Prediction of potential miRs targeting at the 3′-UTR of Cx43 gene (**A**–**C)**. Prediction of the potential miRs targeting at the 3′-UTR of Cx43 gene in human (**A**), mouse (**B**) and rat (**C**). (**D**) The conserved miRs targeting at the 3′-UTR of human Cx43 gene. (**A**–**C**) The underlined numbers indicated the location of the miR-targeting sites in the 3′-UTR of Cx43 gene.

### Identification of multiple miRs in regulating Cx43 expression

To observe the regulatory role of the aforementioned miRs on Cx43 expression, we synthesized and transfected those miRs into MDA-MB-231 cells, a highly invasive human breast cancer cell line [21]. We demonstrated that transfection of *miR-1*, *miR-206*, *miR-200a*, *miR-381*, *miR-23a/b* and *miR-186* notably suppressed the mRNA levels of human Cx43 respectively (Figure 2A). Likewise, the protein levels of Cx43 in MDA-MB-231 cells were also significantly inhibited by the treatment of aforementioned miRs (Figures 2B–2C).

**Figure 2:**
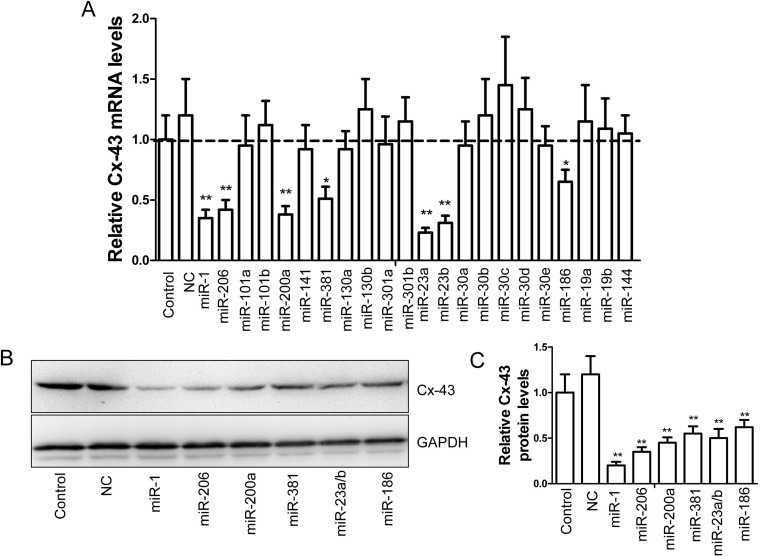
Identification of several miRs in regulating Cx43 expression (**A**). The mRNA levels of Cx43 in the MDA-MB-231 cells transfected with different miRs (100 nM), control miR (NC, 100 nM) or mock as control for 24 h (*n*=5, *P<0.05 and **P<0.01). (**B**) Immunoblotting assay of Cx43 protein in the MDA-MB-231 cells treated as described above in (**A**). (**C**). Statistic of the relative protein levels displayed in (**B**) (*n*=3, **P<0.01).

### *miR-200a* directly inhibits Cx43 expression via a canonic-binding element

We predicted the potential binding sties for *miR-23a/b* (1202), *miR-200* (469, 909), *miR-186* (1077, 1673) and *miR-381* (1317) in the 3′-UTR (from 1 to 1723 bp) of human Cx43 gene (Figure 3A). Then, we subcloned the 3′-UTR (from 1 to 1723 bp) or the ones with mutated miR-binding sites into a miR reporter plasmid to study the role of *miR-23a/b*, *miR-200*, *miR-186* and *miR-381* in Cx43 gene expression (Figure 3B). As expected, *miR-23a*, *miR-23b* and *miR-200a* dramatically suppressed Cx43 expression activity (Figures 3C–3F). However, mutation of the potential element for *miR-23a* or *miR-23b* could not rescue the inhibitory role of *miR-23a* or *miR-23b* in Cx43 expression (Figures 3C–3D), indicating that *miR-23a/b* did not suppress Cx43 expression in a direct manner via the predicted binding sites. Interestingly, the site-specific mutation at 909, but not 469 in the 3′-UTR of Cx43 gene could fully rescue the inhibitory effect of *miR-200a* on Cx43 gene expression (Figures 3E–3F). Unexpectedly, *miR-186* and *miR-381* could not suppress the wt or mutated reporter gene activity (Figures 3G–3H), indicating that those two miRs-inhibited Cx43 gene expression was not in a 3′-UTR dependent manner. Therefore, among the investigated miRs, we identified *miR-200a* as a novel and direct suppressor of Cx43 gene.

**Figure 3:**
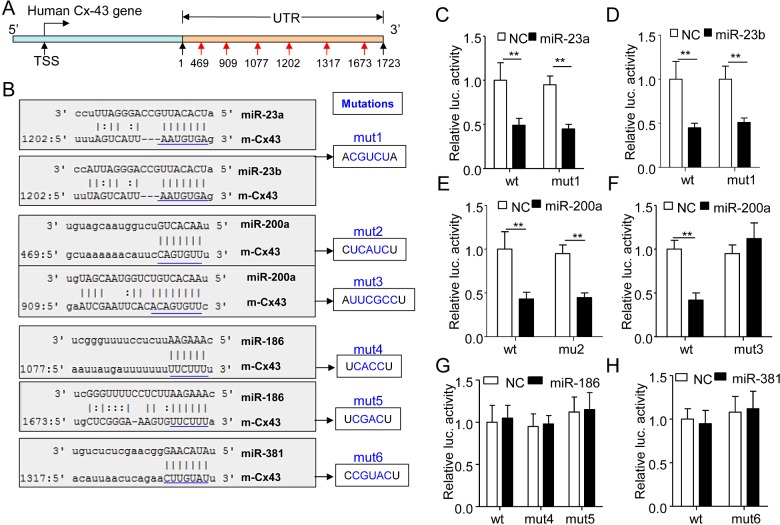
*miR-200a* suppressed human Cx43 gene expression via directly targeting at a binding site locating at 909 in the 3′-UTR of human Cx43 gene. (**A**) The schematic diagram of potential binding sites for functional miRs in the 3′-UTR of human Cx43 gene. (**B**) The 3′-UTR of human Cx43 gene containing the wt or mutated binding sequences of potential miRs was subcloned into a miR reporter vector to form the wt or mutated (mut1–mut6) reporter genes as indicated. (**C**). The wt or mut1 reporter plasmid (0.4 μg/ml) was co-transfected with the control miR (NC, 100 nM) or *miR-23a* (100 nM) into the MDA-MB-231 cells for 24 h and then the cells were harvested for luciferase assay (*n*=5, **P<0.01). (**D**) The luciferase activity of the MDA-MB-231 cells co-transfected with the reporter plasmids wt or mut (0.4 μg/ml) plus NC or *miR-23b* (NC, 100 nM) for 24 h (*n*=5, **P<0.01). (**E**) The luciferase activity of the MDA-MB-231 cells co-transfected with the wt or mut2 (0.4 μg/ml) plus NC or *miR-200a* (NC, 100 nM) for 24 h (*n*=5, **P<0.01). (**F**) The reporter plasmids wt or mut3 (0.4 μg/ml) were co-transfected with the NC or *miR-200a* (100 nM) into MDA-MB-231 cells for 24 h and then the cells were subjected to luciferase assay (*n*=5, **P<0.01). (**G**) The reporter plasmids wt, mut4 or mut5 (0.4 μg/ml) were co-transfected with the NC or *miR-186* (100 nM) into MDA-MB-231 cells for 24 h before cell collection and luciferase assay (*n*=5). (**H**) The luciferase activity of the MDA-MB-231 cells co-transfected with the reporter gene wt or mut6 (0.4 μg/ml) plus the NC (100 nM) or *miR-381* (100 nM) for 24 h (*n*=5).

### Overexpressing Cx43 in human breast cancer cells

The human Cx43 gene consists of 1149-bp CDS Exons and a 1723-bp 3′-UTR, in which we identified a canonic-binding site for *miR-200a* at 909-bp (Figure 4A). We subcloned the human Cx43 CDS Exons only or Cx43 CDS Exons plus UTR into pCDNA3.1 vector to construct human Cx43 overexpression plasmids pCDNA-Cx43Δ or pCDNA-Cx43, respectively (Figure 4B). The latter construct-mediated Cx43 overexpression was supposed to be inhibited by *miR-200a* via the 3′-UTR. As expected, the western blotting assay indicated that both the pCDNA-Cx43Δ and the pCDNA-Cx43 plasmids could increase the expression of Cx43 in a dose-dependent manner (Figure 4C). In addition, the pCDNA-Cx43, but not pCDNA-Cx43Δ-mediated Cx43 overexpression was attenuated by *miR-200a* transfection (Figure 4D).

**Figure 4:**
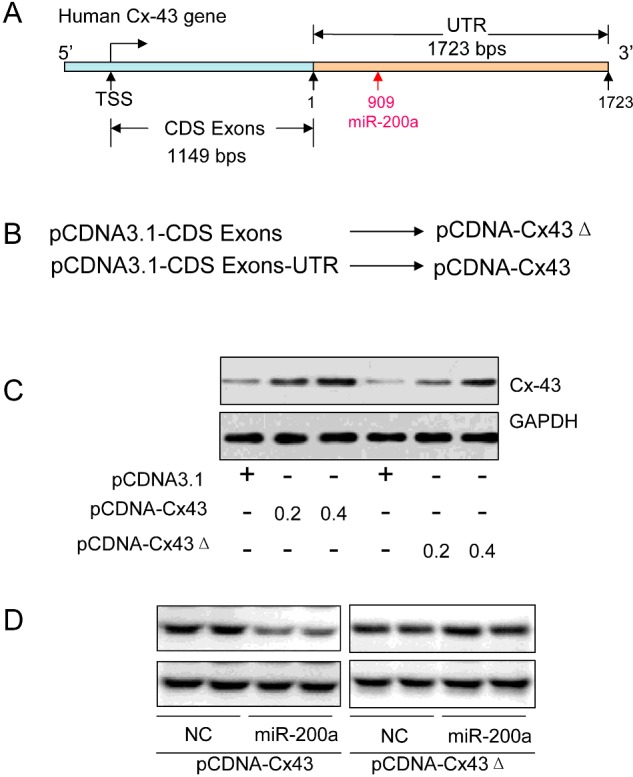
Overexpressing human Cx43 proteins in MCF-7 cells (**A**) The schematic diagram of the human Cx43 gene structure containing the 1149-bp CDS Exons and 1723-bp 3′-UTR. (**B**) The pCDNA-Cx43, a human Cx43 protein overexpression plasmid, was obtained by subcloning the 1149-bp CDS Exons plus 1723-bp 3′-UTR into the pCDNA3.1 vector. The pCDNA-Cx43Δ construct was obtained by subcloning the 1149-bp CDS Exons only into the pCDNA3.1 vector. (**C**) Immunoblotting assay of human Cx43 in the MCF-7 cells transfected with different doses of pCDNA-Cx43 (0.2 or 0.4 μg/ml), pCDNA-Cx43Δ (0.2 or 0.4 μg/ml) or pCDNA3.1 vector as control for 24 h. (**D**) Western blotting assay of human Cx43 in the MCF-7 cells co-transfected with pCDNA-Cx43 (0.4 μg/ml) or pCDNA-Cx43Δ (0.4 μg/ml) plus NC (100 nM) or *miR-200a* (100 nM) for 24 h.

### *miR-200a*/Cx43 axis-mediated migration of breast cancer cells

We further observed whether *miR-200a* could regulate cancer cell migration, since Cx43 was an important contributor to the metastasis of breast cancer [6,24,25]. MCF-7 is a non-aggressive breast cancer cell line. The transwell assay revealed that overexpression of Cx43 in MCF-7 cells with pCDNA-Cx43 transfection potentiated the migration activity and additional *miR-200a* treatment notably prevented this effect (Figures 5A–5B). However, the pCDNA-Cx43Δ transfection-induced migration activity in MCF-7 cells could not be attenuated by *miR-200a* treatment (Figures 5C–5D). MDA-MB-231 is an aggressive breast cancer cell line. The migration activity of these cells could be attenuated by the treatment of *miR-200a* or *miR-1* (Figures 5E–5F). In consistence, both *miR-200a* and *miR-1* were direct suppressors of Cx43. Therefore, we concluded that *miR-200a* could inhibit the migration activity of breast cancer cells through suppression of Cx43 expression.

**Figure 5:**
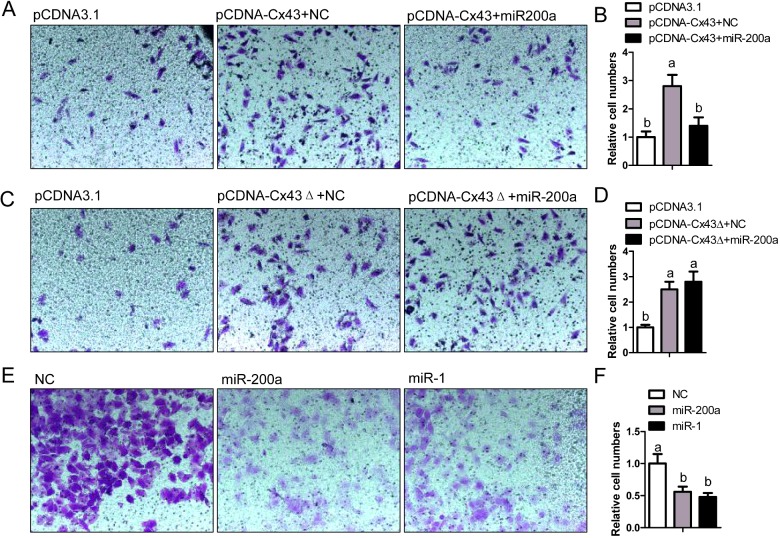
*miR-200a*/Cx43 axis regulated migration activity of breast cancer cells (**A**) Migration activity of MCF-7 cells transfected with pCDNA3.1 (0.4 μg/ml), pCDNA-Cx43 (0.4 μg/ml) plus NC (100 nM) or pCDNA-Cx43 (0.4 μg/ml) plus *miR-200a* (100 nM) was measured by transwell assay. (**B**) Relative transmitted cell numbers in the transwell assay in (**A**). (**C**) Transwell assay of MCF-7 cells transfected with pCDNA3.1 (0.4 μg/ml), pCDNA-Cx43Δ (0.4 μg/ml) plus NC (100 nM) or pCDNA-Cx43Δ (0.4 μg/ml) plus *miR-200a* (100 nM). (**D**) Relative transmitted cell numbers in the transwell assay in (**C**). (**E**) Migration activity of MDA-MB-231 cells transfected with NC (100 nM), *miR-200a* (100 nM) or *miR-1* (100 nM) was measured by transwell assay. (**F**) Relative numbers of migrated cells in the transwell assay in (**E**). (**B**, **D** and **F**) Values not sharing a common superscript letter differ significantly (*n*=5, *P*<0.05).

### Decreased *miR-200a* and elevated Cx43 expression in metastatic breast cancer tissues

Given the regulatory role of *miR-200a*/Cx43 axis in the migration activity of breast cancer cells, we further observed the expression of *miR-200a* and Cx43 in 20 cases of primary and metastatic breast cancer tissues in human patients. We demonstrated that the levels of *miR-1* (Figure 6A) and *miR-200a* (Figure 6B) were significantly decreased, whereas the expression of Cx43 mRNA (Figure 6C) was notably elevated in the metastatic breast cancer tissues compared with the primary ones. Those results confirmed the inhibitory role of *miR-200a* in Cx43 expression and indicated the possible pathophysiological effects of *miR-200a*/Cx43 axis in metastatic breast cancer.

**Figure 6:**
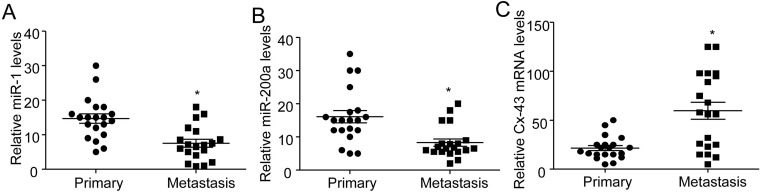
Decreased levels of *miR-200a* and *miR-1* as well as elevated mRNA expression of Cx43 in the metastatic breast cancer tissues in patients (**A** and **B**). Reduced levels of *miR-1* (**A**) and *miR-200a* (**B**) in the metastatic breast cancer tissues compared with the primary ones (*n*=20, *P<0.05). (**C**) Relative Cx43 mRNA levels in the primary and metastatic breast cancer tissues. (*n*=20, *P<0.05)

## DISCUSSION

In the present study, we are the first to identify *miR-200a* as a novel suppressor of Cx43 gene by directly targeting in the 3′-UTR of Cx43 gene. Meanwhile, we also found that *miR-1*, *miR-206*, *miR-381*, *miR-23a*, *miR-23b* and *miR-186* could also inhibit human Cx43 expression in mRNA and protein levels. We did not further explore the targeting sequences of *miR-1* and *miR-206* in human Cx43 gene, since a previous study had reported the direct interaction between those two miRs and Cx43 gene [23].

With regard to *miR-23a* and *miR-23b*, we did not find their potential binding elements in the 3′-UTR of human Cx43 gene, although both miRs exerted a dramatically inhibitory role in Cx43 gene expression. We presumed that *miR-23a* and *miR-23b* might directly act on Cx43 gene via a non-canonic-binding site in the 3′-UTR, but not through the one we predicted or that those two miRs might indirectly suppress human Cx43 gene expression via regulating some other miRs which could interact with the 3′-UTR of human Cx43 gene.

As to *miR-186* and *miR-381*, both of them could inhibit the mRNA and protein levels of human Cx43, but they exerted no significant effects on the activity of Cx43 3′-UTR reporter gene. Those results indicated that *miR-186* and *miR-381* might not act directly on the 3′-UTR of human Cx43 gene. They might regulate Cx43 expression by acting on some transcription factors which could target Cx43 gene promoter. Therefore, the present study uncovered a complex network of miRs in the regulation of human Cx43 gene expression. The above presumptions need to be verified by further experiment in future.

Cx43 expression was associated with increased malignancy in multiple cancers [26–28]. Especially, a previous study reported that *miR-206* inhibited the migration and invasion of breast cancer by targeting at Cx43 [25]. Those observations indicated that the miRs we identified in the present study might be functional regulators of the migration and metastasis of breast cancer via regulating Cx43 expression. Our results verified these notions: (1) overexpressing Cx43 in a non-aggressive breast cancer cell line MCF-7 enhanced the cell migration activity; (2) Additional treatment of *miR-200a* could fully prevent Cx43-induced cell migration activity in MCF-7 cell; (3) Treatment of *miR-200a* or *miR-1*, both of which were direct suppressors of Cx43, could attenuate the migration activity of an aggressive breast cancer cell line MDA-MB-231.

To further correlate the expression of *miR-200a* and Cx43 to the physiopathology of breast cancer *in vivo*, we collected the primary and metastatic breast cancer tissues in clinics. We found the notably decreased levels of *miR-1* and *miR-200a*, as well as the increased mRNA expression of Cx43 in the metastatic breast cancer tissues compared with the primary cancer tissues. These findings indicated that the *miR-1*/Cx43 or *miR-200a*/Cx43 axis might be promising targets for the diagnosis and therapy of metastatic breast cancer.

In conclusion, we identified *miR-200a* as a novel suppressor of Cx43 gene by directly targeting in the 3′-UTR. The *miR-200a*/Cx43 axis regulated the migration activity of the breast cancer cells.

## Abbreviations

CDS: coding sequence
Cx43: connexin 43
DMEM: Dulbecco's Modified Eagle's Medium
ERα: estrogen receptor α
HER-2: human epidermal growth factor receptor-2
miR: miRNA
PR: progesterone receptor
UTR: untranslated region
wt: wild type
